# Current News

**Published:** 1896-03

**Authors:** 


### Current News.

### WOMAN'S DENTAL ASSOCIATION.

THE Woman's Dental Association held its regular meeting at the office of the president, Dr. Anna T. Focht, Broad Street and Columbia Avenue, January 4, 1896.

The essayist, Dr. Ellen Macmurray, of Chester, Pennsylvania, was not able to be present, so that time was given to incidents of office practice.

A demonstration of porcelain work was given by Dr. Focht.

Essayist on the list for February 1, Dr. Henry I. Dorr.

EMILY W. WYETH,
*Recording Secretary.*

3940 FAIRMOUNT AVENUE.

